# Lipid-Based Nanovesicular Drug Delivery Systems

**DOI:** 10.3390/nano11123391

**Published:** 2021-12-14

**Authors:** Tania Limongi, Francesca Susa, Monica Marini, Marco Allione, Bruno Torre, Roberto Pisano, Enzo di Fabrizio

**Affiliations:** Department of Applied Science and Technology, Politecnico di Torino, Corso Duca degli Abruzzi 24, 10129 Turin, Italy; francesca.susa@polito.it (F.S.); monica.marini@polito.it (M.M.); marco.allione@polito.it (M.A.); bruno.torre@polito.it (B.T.); roberto.pisano@polito.it (R.P.); enzo.difabrizio@polito.it (E.d.F.)

**Keywords:** lipid vesicles, niosomes, proniosomes, ethosomes, transferosomes, pharmacosomes, ufasomes, phytosomes, catanionic vesicles, extracellular vesicles

## Abstract

In designing a new drug, considering the preferred route of administration, various requirements must be fulfilled. Active molecules pharmacokinetics should be reliable with a valuable drug profile as well as well-tolerated. Over the past 20 years, nanotechnologies have provided alternative and complementary solutions to those of an exclusively pharmaceutical chemical nature since scientists and clinicians invested in the optimization of materials and methods capable of regulating effective drug delivery at the nanometer scale. Among the many drug delivery carriers, lipid nano vesicular ones successfully support clinical candidates approaching such problems as insolubility, biodegradation, and difficulty in overcoming the skin and biological barriers such as the blood–brain one. In this review, the authors discussed the structure, the biochemical composition, and the drug delivery applications of lipid nanovesicular carriers, namely, niosomes, proniosomes, ethosomes, transferosomes, pharmacosomes, ufasomes, phytosomes, catanionic vesicles, and extracellular vesicles.

## 1. Introduction

Despite relevant technological improvements, developing an effective and safe drug can be a complex, low success rate, time-consuming, and costly practice. As reported on the official webpage of the US Food and Drug Administration (FDA), only a small number of treatment tools (active molecules, nanoparticles, and so on) proposed as skilled medical products, after early testing, result as eligible for further study. In 2020, the FDA’s Center for Drug Evaluation and Research (CDER) authorized 53 novel therapeutics, more than double what happened from 2006–2010. More in details considering the three major therapeutic areas, the new approved drugs are 18 (34%) cancer products, 8 (15%) Neurology products, and 6 (11%) infectious diseases treatments. The average projected peak sales of a just approved drug in 2020 was about USD 700 million, and this is below a long-term average of USD 1.3 billion and a median of USD 500 million [1].

The constant development of technologies and materials resulting from the collaboration between sectors such as bioengineering, physics, chemistry, materials science, pharmacology, and not least medicine, has allowed the advancement of increasingly efficient drug delivery tools. Researchers and clinicians from all over the world daily pursue the design and implementation of increasingly personalized, safe, and cheap care solutions as new pharmacologically active molecules and nanoparticles. Recently, the application of nanoparticles (NPs) has been established to develop drug delivery efficiency. Nanomaterials generally refer to a material characterized by having at least one dimension in the nanometer scale (1–100 nm) [2], include nano-drug delivery systems that thanks to their morphological, optical, mechanical, and electrical characteristics can improve drugs’ stability and solubility by extending their blood circulation time and enhancing their delivery efficiency.

Metallic, polymeric, organic, and inorganic nano scaled materials including dendrimers, nanotubes micelles, and quantum dots (QDs) have been recently assessed as drug delivery carriers (DDC) [3,4,5].

Among the already numerous nanoscale DDCs, nanovesicles represent highly-promising effective approaches to setting up therapies against cancer, inflammation infection, and degenerative disorders.

In this review, we described the most modern lipid-based nanovesicular systems, whether they are of biological or synthetic origin, used for the most distinct biomedical and clinical applications. We left liposomes, already the subject of numerous and recent scientific publications, out of the topics covered in this review, to make room for other lipidic nanovesicles, perhaps less known, but increasingly the target of studies for drug delivery applications such as niosomes, proniosomes, ethosomes, transferosomes, pharmacosomes, ufasomes, phytosomes, and catanionic vesicles. Last, but certainly not least, the type of Lipid NanoVesicles (LNV) discussed in this review are the extracellular vesicles (EVs) and their increasingly wide application as DDC of inorganic NPs, drugs, and nucleic acids. For each type of LNV category covered by the discussion, we provided an updated table listing in a very detailed way, the biochemical composition of each vesicle, its cargo, and the application for which it has been designed and studied referring to the in vitro and in vivo drug delivery applications of the last 10 years.

## 2. Proniosomes and Niosomes

**Figure 1:**
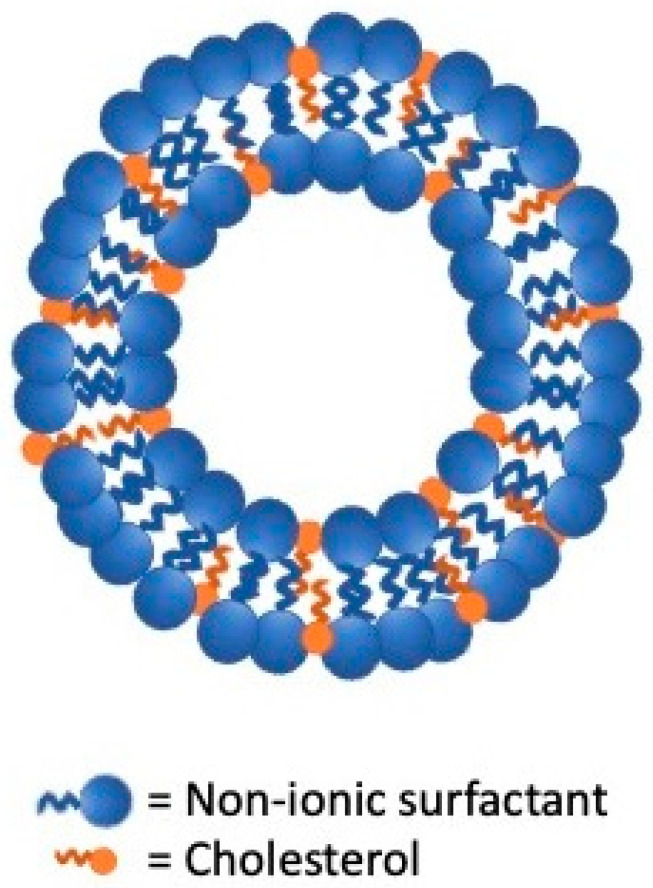
Structure of proniosomes lipid vesicular systems.

**Figure 2:**
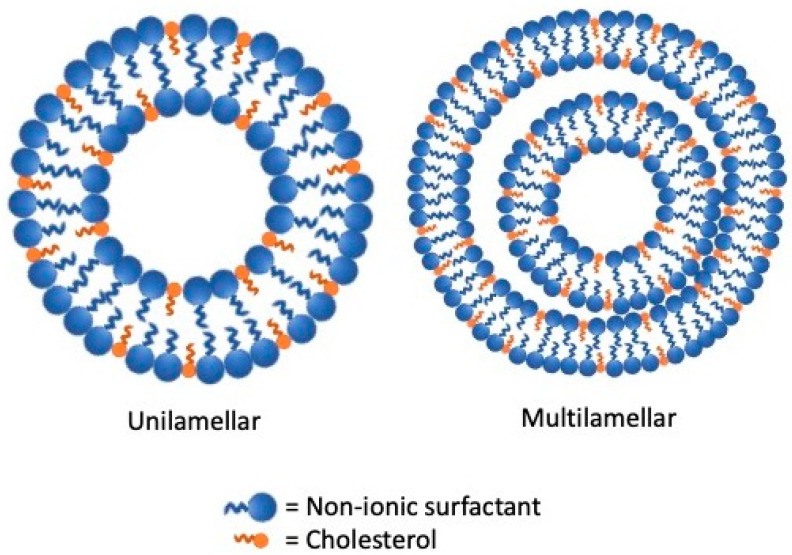
Structure of niosomes lipid vesicular systems.

Niosomes and proniosomes are LNV systems characterized by distinctive amphiphilic structures able to improve poorly soluble drugs bioavailability. Their uniqueness is in having a nonionic surfactant backbone while their multilamellar and unilamellar vesicles structures appear similar to that of liposomes [6] (Figure 1 and Figure 2).

It is assumed that lipophilic molecules are confined within the lipid bilayers while the hydrophilic ones are retained in the niosomes’ aqueous partitions. This efficient compartmentalization improves the stability of the enclosed drugs preventing their chemical and enzymatic degradation [7]. Proniosomes are nonionic dehydrated structured provesicles in the powdered form or in the gel states. Provesicles are water soluble dry free-flowing granular products that can be immediately rehydrated before use avoiding many issues related to aqueous vesicular dispersions. Proniosomes and niosomes can be produced by using cholesterol, non-ionic surfactants (Tween 20, 40, 80, Span 20, 40, 60, 80, 85), solvents as chloroform and methyl and ethyl alcohols and lecithin. Usually, surfactants utilized to produce niosomes and proniosomes are characterized by low aqueous solubility but Tween can be successfully used to produce micelles on hydration [8].

Niosomes are similar to liposomes, but they are cheaper, exhibit a higher stability, encapsulation efficiency, and permeability for small molecules, avoid the degradation of phospholipids by oxidation, and are easier to store and handle. Indeed, niosomes display some drawbacks, such as aggregation, fusion, and leakage of drugs, while proniosomes can overcome these issues contrasting leakage, aggregation, or hydrolysis of drugs while optimizing their storage and biodistribution, adding the possibility of sterilization, room temperature storage, and being rehydrated instantly to create niosomes [9].

Proniosomes have several pluses over niosomes, contrasting leakage, aggregation, or hydrolysis of drugs while optimizing their storage and biodistribution.

Although the first applications of non-ionic surfactant nanovesicles were cosmetic ones [10,11], in Table 1 and Table 2, we report the numerous and recent drug delivery applications for proniosomes and niosomes, respectively.

Thanks to their capability to store and deliver both hydrophilic and hydrophobic medications through topical, oral, transmucosal, pulmonary, ocular, and parenteral/intravenous administration, niosomes and proniosomes are increasingly used as vaccines and treatments for infection, inflammation, cancer, and many other acute or chronic diseases.

## 3. Ethosomes

**Figure 3:**
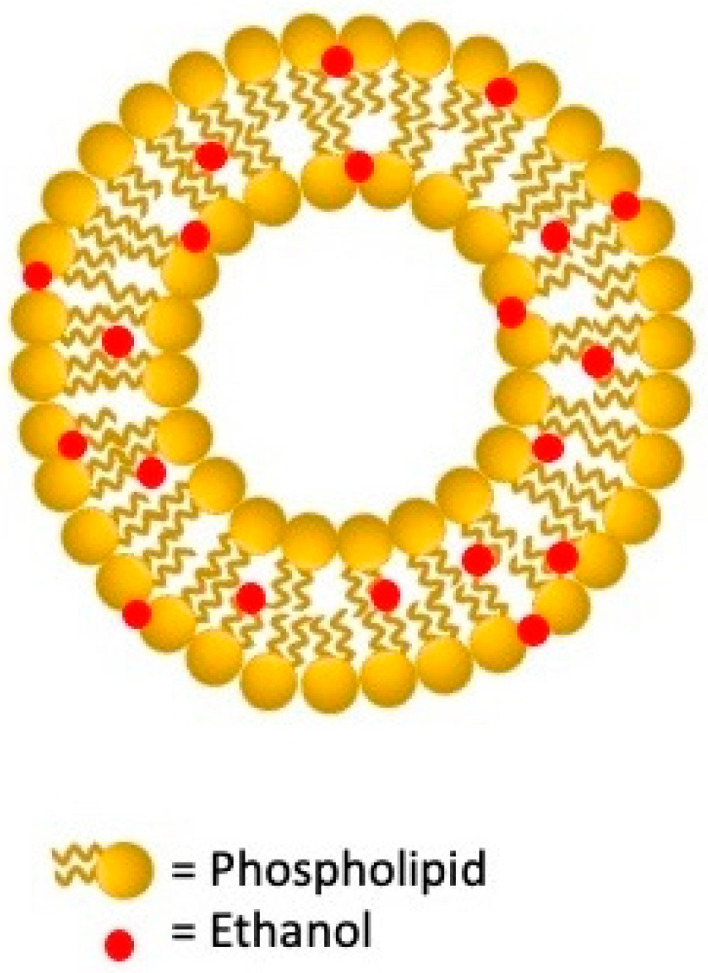
Schematic structure of ethosome lipid vesicular system.

Ethosomes were designed and developed in 2000 by Touitou et al. [108] as an advanced noninvasive passive lipid-based delivery system. As represented in Figure 3, these carriers are lipid bilayers composed of phospholipids, water, and high concentrations of ethanol which gives them remarkable transdermal permeability skills. Ethanol and lipid molecules act in the polar head group region increasing membrane fluidity and permeability. Ethosomes have significantly improved skin delivery, carrying the active compounds in the deeper layers of the skin in occlusive and non-occlusive conditions. In addition, they display high deformability, encapsulation efficiency, stability, biocompatibility, and a negative charge due to ethanol that leads to small vesicles size, enhancing the bioavailability of the compounds. Despite these advantages, there are some drawbacks caused by the volatile nature of ethanol, such as problems related to system instability, drug leakage, and skin irritation [109]. These vesicles are successfully used for topical administration of a considerable variety of drugs such as antifungals, antivirals, antibiotics, anti-inflammatories, and many others as detailed in Table 3.

## 4. Transfersomes

Many drug delivery systems have been designed over the past decades for transdermal administration, which offers many advantages over other routes thanks to its capability of escaping presystemic metabolism, tune drug release reducing variation in drug levels, enhancing pharmacological response. Compared to most other transdermal delivery methods including chemical permeation enhancers, sonophoresis, microneedles, lipid vesicles thanks to their distinctive composition can transport both hydrophilic and lipophilic drugs [140].

**Figure 4:**
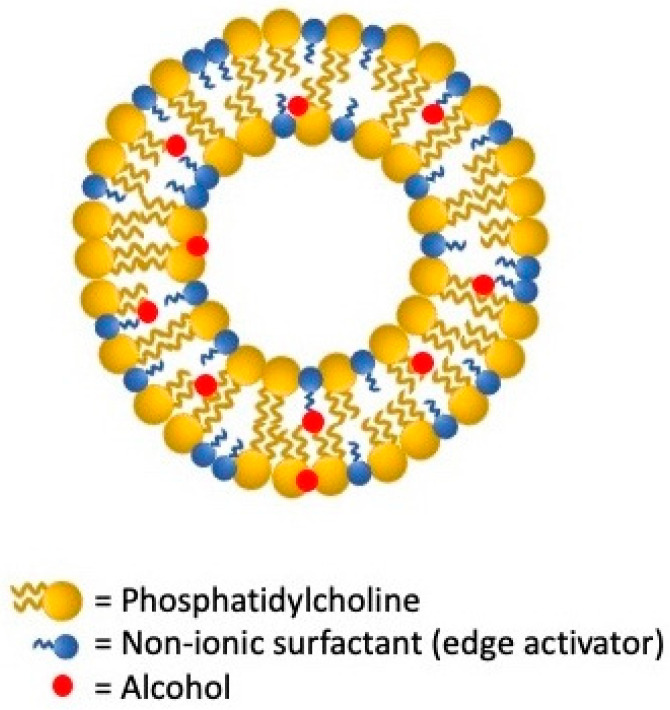
Schematic structure of transfersomes lipid vesicular system.

Among the LNV, transfersomes, first proposed in the early 1990s, are ultra-deformable elastic vesicles successfully employed as a non-occluded method able to permeate skin through the stratum corneum reaching the dermis and blood circulation [141]. As schematized in Figure 4, they are firstly characterized by an aqueous core enclosed by a lipid bilayer of amphipathic constituent as phosphatidylcholine, lecithin, or a mixture of lipids. In addition to a very low percentage of alcohol (3–10%), they are made with 10–25% of bilayer-softening complexes, surfactants, or edge activators as Tweens, Spans, sodium cholates, and deoxycholate. The appropriate phospholipids/surfactants ratio tunes transferosomes’ membrane elasticity reducing vesicles’ rupture chances through the skin [142,143]. By having edge activators in their structure, thanks to their remarkable elastic properties, transfersomes defeat many main liposomes’ weaknesses resulting in more apt to squeeze themselves through the skin barrier [144]. Despite these advantageous properties, transfersomes exhibit also some drawbacks, i.e., chemical instability due to the oxidative degradation and expensiveness in the precursors and manufacturing [143].

Thanks to their enhanced skin-penetration abilities, transfersomes are competent to set up skin drug storage area for continuous therapeutic molecules delivery releasing low, as well as high, molecular weight drugs as antioxidants, chemotherapy, anti-Inflammatory, and corticosteroids (Table 4).

## 5. Pharmacosomes

**Figure 5:**
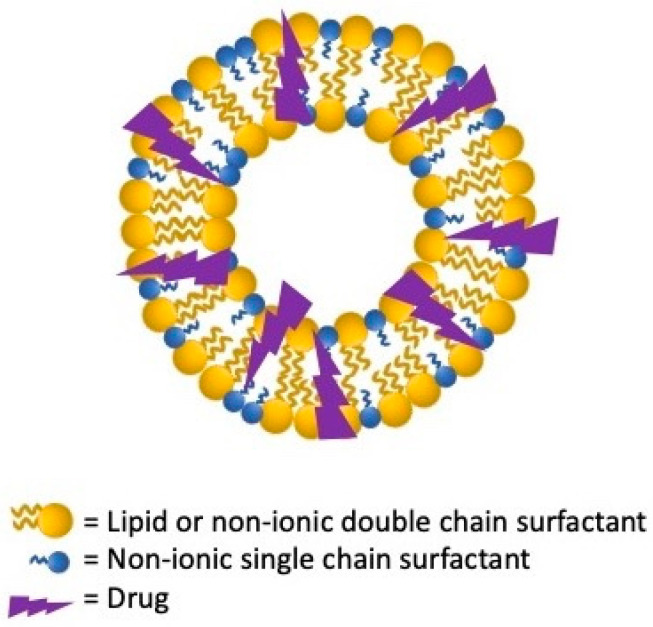
Schematic structure of pharmacosomes lipid vesicular system.

The name pharmacosomes refers to the amphiphilic, zwitterionic, stoichiometric complexes of polyphenolic compounds with phospholipids, as schematized in Figure 5. The success in the use of pharmacosomes is explained by the surface and bulk interactions of lipids with drugs since the latter possess an active hydrogen atom as –OH, -COOH, -NH_2_, which can be esterified to the lipid causing an amphiphilic compound [166,167].

The use of pharmacosomes in drug delivery has several advantages over that of other vesicles such as niosomes, transferosomes, and liposomes. More in detail, any active molecules in which a carboxyl group is present can be esterified without a spacer chain as opposed to those characterized by the presence of amino or hydroxyl groups which, in order to be esterified, require spacer groups. Pharmacosomes design is based on the phospholipids/water superficial and bulk interaction; the drug molecule and the connected lipid molecule, respectively, behave like the polar head group and the lipidic chain giving the molecule an amphipathic character. Thanks to their hydrophilic and lipophilic properties, these lipid LNV improve drugs’ dissolution in gastrointestinal fluid, increasing the bioavailability of low soluble treatments avoiding leak and rupture release [168,169]. Pharmacosomes’ in vivo pharmacokinetic performances are conditioned by vesicles’ dimension, by the drug molecule’s functional groups, by the lipids’ fatty acid chain length, and, last but not least, by the spacer groups’ availability. The high tunability of each of the components listed above makes these types of vesicles excellent candidates for the effective delivery of a wide range of active molecules including anti-cancer and anti-inflammatory remedies (Table 5) [170].

Among the few limitations relating to the use of pharmacoses, reference should be made to their susceptibility to hydrolyzation, fusion, or aggregation during storage or engineering processes [171,172].

**Table 5:** Pharmacosomes’ drug delivery applications.

Composition	Cargo	Application	Reference
Doxifluridine and DOTAP	miR-122	Treatment of hepatocellular carcinoma	[173]
Etoricoxib and phosphatidylcholine		Rheumatoid arthritis treatment	[174]
Folic Acid-Modified 2-Deoxyglucose and amino ethanol		Targeting anti-tumor therapy	[175]
Ibuprofen and Phosphatidylcholine from soy		Anti-inflammatory	[176]
Levodopa, egg lecithin and chitosan		Parkison’s treatment	[177]
Naproxen and soy lecithin		Rheumatoid arthritis treatment	[178]
Rosuvastatin, soy lecithin and cholesterol		Hyperlipidemia treatment	[179]

## 6. Ufasomes

**Figure 6:**
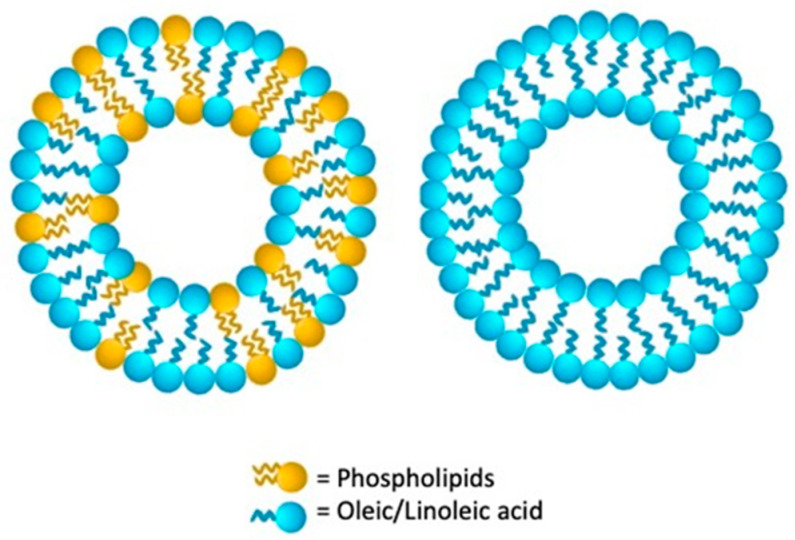
Schematic structure of ufasomes lipid vesicular system.

Unsaturated fatty acid vesicles preparation, more commonly known as ufasomes, was first reported in 1973 by Gebicki and Hicks [180]. In a controlled pH range, from 7 to 9, they are a closed lipid bilayered suspension, made from unsaturated fats and their ionized species. In detail, fatty acid molecules’ hydrocarbon tails are directed toward the deeper membrane layer while the carboxyl heads are in contact with water [181], as schematized in Figure 6. Oleic and linoleic acid (cis, is-9,12-octadecadienoic acid), the major ufasomes’ constituents, confer to these nanovesicles a more versatile nature than that of the other LNV, by ranking them between different nanosystems formed from double-chain amphiphiles and from single-chain surfactants micelles. Their biochemical composition makes them easily to assemble and real biocompatible [182,183]. By enhancing ufasomes stability with the identification of the appropriate fatty acid, pH range, and lipoxygenase amount, increasingly targeted and effective drug delivery solutions are being developed (Table 6).

## 7. Phytosomes

**Figure 7:**
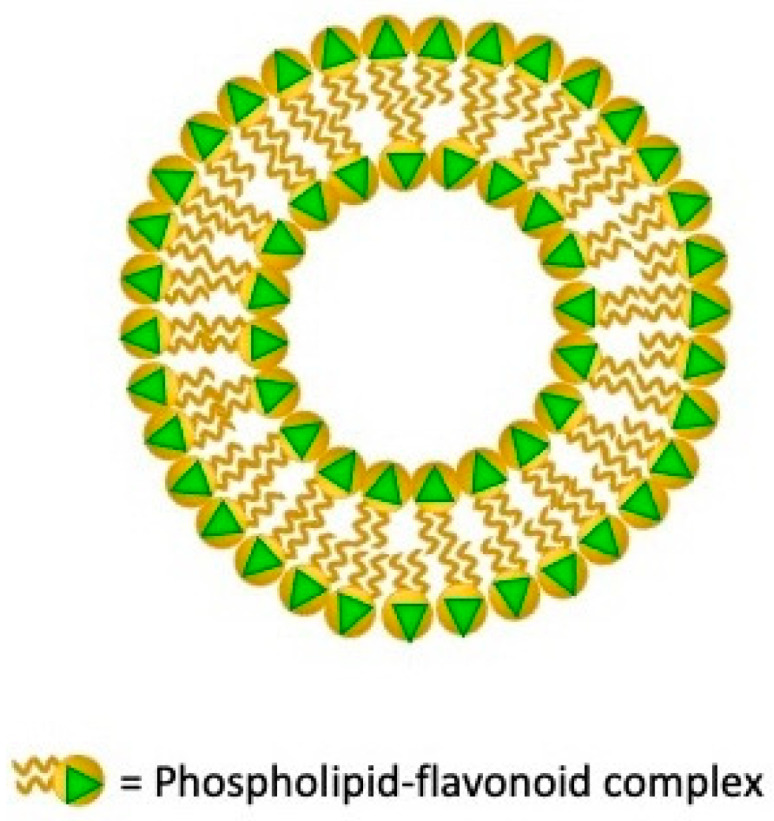
Schematic structure of phytosomes lipid vesicular system.

Although for a long time phyto-pharmaceuticals have a prominent position in the therapeutic scene, it should be emphasized how phyto-active constituents as phenolics, flavonoid, and terpenoids demonstrate considerable in-vitro bio-action but are still characterized by low in-vivo effectiveness due to their high molecular weight, low lipid solubility, and bioavailability [188]. Phytosomes nanovesicles originating by Phyto-Phospholipid Complex (PPC), have been developed as a capable strategy to improve natural drugs delivery and bioavailability. PPCs originate by the phospholipids’ polar head and active constituents’ interactions. The two long fatty acid chains do not take part in the formation of the complex, they can interchange encapsulating the polar region of complexes originating a lipophilic side when resuspended in water (Figure 7) [189].

Phytosomes have many structural and functional aspects in common with liposomes and tranferosomes such as the capability to improve the solubility of weakly soluble polyphenolic phytochemicals. Otherwise, phytosomes and transferosomes are more stable than liposomes in 4 °C and 25 °C aqueous media up to three months since liposomes should be freeze dried to preserve their stability. Phytosomes, as well as transferosomes, exhibit superior dermal penetration properties leading noticeable accumulation in the epidermis and dermis. Since the phytosomes configuration is grounded on the H-bond interaction between the phospholipid molecules’ polar moiety and the phytoconstituents, the laded compounds permanence is higher than in other lipid nanovesicles [190]. The numerous and very recent drug delivery applications collected in Table 7 show how phytosome nanotechnology will definitely get more efficient the ways of bioactive phytochemicals therapeutic and aesthetic delivery counteracting the bottlenecks of the low absorption and poor penetration rate across biological barriers improving herbal-originated compounds pharmacodynamic and pharmacokinetic and assets [190].

## 8. Catanionic Vesicles

**Figure 8:**
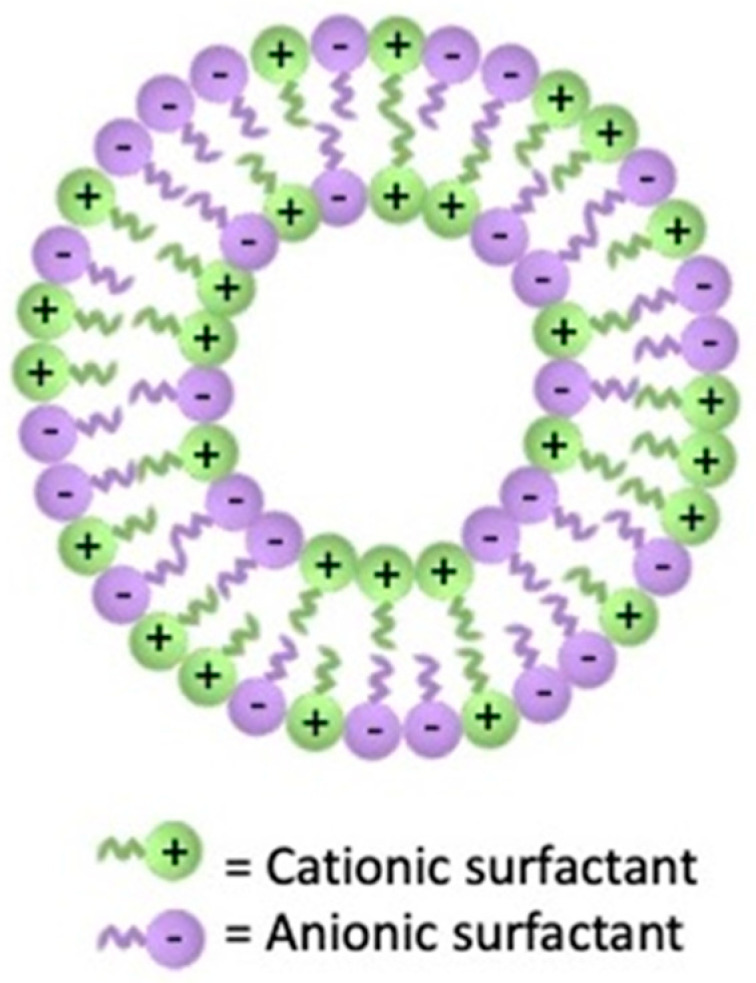
Schematic structure of catanionic vesicles.

An innovative class of biocompatible and biodegradable drugs lipidic nanovehicle is represented by the catanionic vesicles for their capability to improve the stability and cellular uptake of a wide range of active molecules [215]. These hybrid nanovesicles spontaneously form when unequal amounts of cationic and anionic single-tailed surfactants are dispersed in water [216] (Figure 8).

These nanovesicles are produced by using easily accessible cheap surfactants and, in comparison with phospholipid vesicles, are thermodynamically advantaged in terms of colloidal stability. Alkyl ammonium bromide and gemini surfactants such as bis-quaternary ammonium salts have been used for catanionic vesicles production; however, since they are cytotoxic and not biodegradable, the conjugation with safer molecules is being successfully considered [217]. Their low production costs, higher stability and drug loading capability, together with the fact that they suffer less from ruptures and pressure drops make them excellent drug delivery vehicles for vaccination and anti-microbial, cancer, and inflammatory applications (Table 8). Thus, although catanionic vesicles have a huge applicability in biomedicine, they can suffer safety problems due to their eventual low bio- and emocompatibility. Numerous ongoing researches point to the optimization of their morphology, hydrophobicity, and ionic charge by carefully choosing the proper surfactant and by tuning the anionic/cationic surfactant ratio eventually adding some suited additive [218].

## 9. Extracellular Vesicles

**Figure 9:**
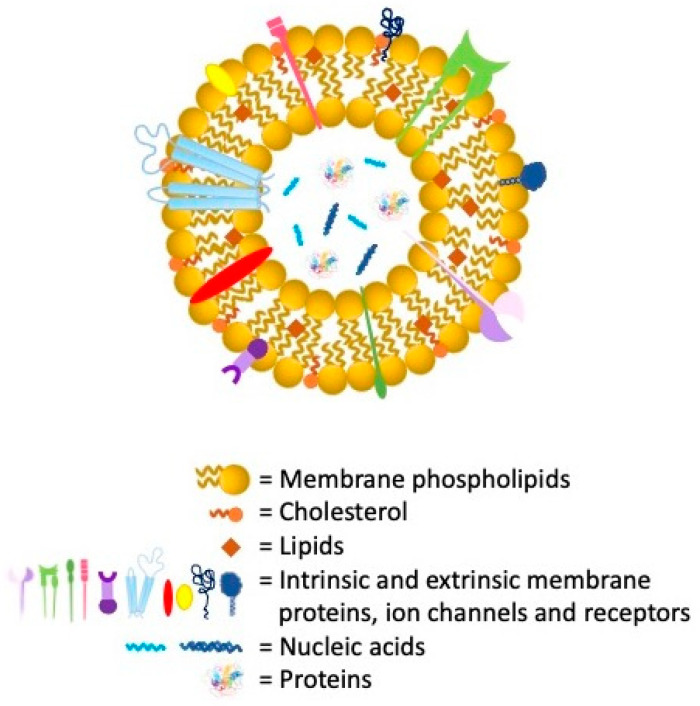
Schematic structure of extracellular vesicles.

The most heterogeneous and versatile class of lipid vesicles is certainly that of extracellular vesicles (EVs) (Figure 9) including apoptotic bodies, microvesicles, and exosomes. These vesicles are ubiquitarian and can be isolated from cells culture media and from all the major biological fluid as urine, plasma, saliva, amniotic and cerebrospinal fluid, semen, among others [242,243,244,245]. Both apoptotic bodies and microvesicles, with dimensions ranging between 500 nm and 2 µm and from 50 nm to 1 µm, respectively, arise from plasma cell membrane outward blebbing and fragmentation. On the other side, exosomes, deriving from the endocytic pathway, have diameters between 30 to 120 nm [246]. Many authors reported about the EVs use in drug delivery since their surface is characterized by antigens, related to the parental cells, able to direct specific homing or targeting phenomena [247]. Although the EVS, as the main physio-pathological intracellular communication mediators, are already in origin able to transport miRNA, proteins, and other biological molecules, their morpho-functional and biochemical characteristics make them excellent candidates for post isolation nanotechnological modifications. In the last twenty years, numerous studies show the great potential of these vesicles in both the diagnostic and therapeutic fields [248]. Their high biocompatibility, low immunogenicity coupled with a superior loading capability make them proper tools for post isolation drug delivery load and engineering. In addition to a whole series of chemical or biological functionalization, many studies are referring to the possibility of loading them with cellular organelles such as mitochondria, NPs, drugs, and nuclei acids [249,250,251].

Although the intrinsic complexity related to the EVs’ size and natural (batch-to-batch) heterogeneity makes their drug delivery application much more complex than that with merely synthetic production systems, many exogenous EVs’ active molecules loading methods have been successfully proposed for the clinical EVs’ translation [252] (Table 9).

Many types of cell-derived exosomes, coming from both plant and human eukaryotic cells, have recently been used to successfully encapsulate inorganic NPs. The cargo can be either loaded by treating parental cells or by post EVs isolation engineering [299]. The potential benefits of a wide range of inorganic NPs-loaded EVs have been proven in various drug delivery applications as extensively listed in Table 10.

Since EVs are remarkably involved in genetic information transfer in normal and pathological states [325,326,327], it is not difficult to see their potential as engineered nucleic acids carriers for drug the treatment of ischemic stroke, myocardial infarction [328], traumatic brain injuries [329], and liver fibrosis [330].

The intrinsic properties of EVs such as low immunogenicity and safety make them a suitable candidate for gene cancer therapy with promising advantages with respect to the conventional chemotherapeutic treatments. EVs transfer their RNA or DNA cargo to the target cells with the aim to alter the tumoral genes information and act, e.g., as tumoral suppressors. In addition, the therapeutic properties of EVs-nucleic acids loaded can be further improved by tailoring their surface [331] in order to maximize specificity and successful delivery. In Massaro et al. [332] is reported a list of the ligands used for cancer therapy. Interestingly, attempts to conjugate RNAs to molecules such as cholesterol for EVs surface functionalization were reported [333,334], with the aim to improve loading control and delivery. Therapeutics effects of Plasmid DNA, mRNA, miRNA, and shRNA delivery EV-mediated were reported in Table 11 underlining how gene therapy combined with EVs delivery is a rapidly growing field for safe and effective precision medicine treatments.

## 10. Conclusions

It is well known that liposomes, assumed to be the oldest category of lipidic nanovesicles, have been broadly considered as the major candidates for biomedical and drug delivery applications. Despite their high biocompatibility and the ability to effectively carry both hydrophilic and/or hydrophobic active molecules to the target site, they still suffer some unresolved weaknesses such as brief shelf-life, low colloidal stability, and limited and expensive preparation methods [389]. The development of new drug delivery approaches has significantly boosted the design and the production of the just reviewed non-liposomal lipid nanovesicles. This new cohort of lipid vesicles can complement liposomes as alternative nanovesicular drug delivery systems and although recently implemented, they have all the chances to overspread as successful engineered nanomaterials.

Considering the existent non-liposomal LNV, those collected in this review, given their countless listed applications, have undoubtedly proved to be the most successful ones by reaching clinical use. Surely among the different types of LNV described in this review, those of cellular origin, the extracellular vesicles, are those that could also give future results closer to the needs of personalized medicine therapeutic plans. The possibility of isolating them from the same patient who is going to be treated reduces the likelihood of rejection phenomena both by increasing the compliance of the therapy and by reducing any adverse effects. Therefore, it would be foreseen that very soon, the LNV carrier’s production will scale-up from the lab scale to the industrial one issuing high-quality competitive outcomes.

In this regard, we would like to conclude with an update on the recent and promising use of lipid nanovesicles for the nucleic acids based-vaccine development. This application has been mainly oriented to the oncologic field, but recently, under the pressure of the latest terrible health emergency that has afflicted the entire globe, anti-viral applications have been reported. EV-based vaccines to deliver mRNA coding for specific molecules such as proteins or by the exposure of specific features on EVs surface have been designed. Since 2020, the SARS-CoV-2 pandemic has boosted additional efforts for the successful design of forceful vaccines [332,390]. Leading approved vaccines provide immunization by the viral Spike (S) protein, injected as purified proteins or codified by the administered mRNAs sequences and showing that “mRNA-based vaccines can fill the gap between emerging pandemic infectious disease and a bountiful supply of effective vaccines” [391]. The mRNA-based vaccine BNT162b2 was developed by Pfizer/BioNTech while the mRNA-1273 SARS-CoV-2 vaccine was developed by Moderna [392]. In Tsai et al. [364] was reported another approach for SARS-CoV-2 vaccines: exosomes are used to deliver mRNAs sequences with the aim to express not only the spike protein but also another artificial protein named “LSNME” and containing the viral spike, nucleocapsid, membrane, and envelope proteins. This approach has been tested on mice with promising results and, along with the many other applications reported in this review, confirmed the growing potential of lipid nanovesicles-mediated delivery as an effective tool for the translation of nanotechnology, bioengineering, and nanomaterials studies from research to clinic.

## Figures and Tables

**Table 1:** Proniosomes’ drug delivery applications.

Composition	Cargo	Application	Reference
Cholesterol, Span 60 and maltodextrin	Aceclofenac	Anti-inflammatory in osteoarthritis	[12]
Cholesterol, Span 60, maltodextrin and stearylamine	Acemetacin	Anti-inflammatory	[13]
Cholesterol, lecithin, Span 60 and Span 40	Atenolol	Hypertension treatment	[14]
Cholesterol, lecithin and Tween 80	Atorvastatin calcium	Anti- hyperlipidemic	[15]
Cholesterol, lecithin and Span 40	Boswellic acid	Anti-inflammatory	[16]
Cholesterol, lecithin and Span 60	Caffeine	Migraine treatment	[17]
Cholesterol and Span 60	Cilostazole	Anti-platelet	[18]
Cholesterol, lecithin and Span 60	Clozapine	Treatment of psychiatric disorders	[19]
Cholesterol, lecithin and cremophor RH	Curcumin	Against ocular inflammation	[20]
Cholesterol, Span 60 and Tween 80	Ciprofloxacin	Anti-inflammatory	[6]
Cholesterol, Span 40 TPGS	Docetaxel	Anticancer treatment	[21]
Cholesterol and Span 60	Famotidine	H_2_ receptor antagonist	[22]
Cholesterol, Sorbitol and Span 80	Flurbiprofen	Anti-inflammatory	[23]
Cholesterol and Brij35	D-limonene	Cancer therapy	[24]
Cholesterol, Span 60	Itroconazole	Antimicotic against candida albicans	[25]
Cholesterol, lecithin and cremophor RH 40	Lacidipine	Treatment of hypertension and atherosclerosis	[26]
Cholesterol, Tween 80, sorbitol and sucrose	Letrozole	Breast cancer	[27]
Cholesterol, Span 80 and lecithin	Lignocaine Hydrochloride	Dental anesthesia	[28]
Cholesterol, Span 60 and Tween 60	Lomefloxacin HCl	Treatment of bacterial conjunctivitis	[29]
Cholesterol, lecithin and Lutrol F68	Lornoxicam	Anti-inflammatory for rheumatoid arthritis, osteoarthritis and surgeries	[30]
Cholesterol, lecithin and Span 60	Embelin	Analgesic and anti-inflammatory	[31,32]
Span 40, Span 60, and Brij series 72	Fluconazole	Management of dental pain	[33]
Cholesterol, lecithin and Span 60	Naproxen	Anti-inflammatory	[32]
Cholesterol, Span 60 and maltodextrin	Pentazocine	Management of cancer pain	[34]
Cholesterol, Span 60, maltodextrin, pullulan and DPPH	Resveratrol	Controlling free radicals causing oxidative stress-induced cardiovascular diseases, atherosclerosis, cancer	[35]
Cholesterol, Span 60, lecithin and stearylamine	Risperidone	Treatment of schizophrenia and other psychiatric disorders	[36]
Cholesterol, lecithin and Span 80	Tramadol	Anti-inflammatory and antinociceptive	[37]
Cholesterol, Span 60, lactose and mannitol	Vismodegib	Carrier for the pulmonary route	[38]

**Table 2:** Niosomes’ drug delivery applications.

Composition	Cargo	Application	Reference
Span 60, cholesterol and bile salt	Acetazolamide	Decrease ocular pressure in glaucoma patients	[39]
Span 60, cholesterol, HMPC and carbopol	Acetazolamide and carvedilol	Decrease ocular pression in glaucoma patients	[40]
Cholesterol, lecithin, Span 60 and Tween 60	Acyclovir	Antiviral	[41]
Span60, Cholesterol and DCP or Span60, Cholesterol and TPGS			[42]
Cholesterol and Span 40	Betaxolol	Glaucoma treatment	[43]
Ergosterol, Span 60 and Tween 60	Carum	Anticancer	[44]
Cholesterol and Span/Tween 60	Carvedilol	Congestive heart failure, coronary artery disease, postmyocardial settings	[45]
Bile salt-enriched vesicles, with 20% sodium cholate and 30% sodium taurocholate		Beta receptor blocking activity to preclude angina and cardiac arrhythmias	[46]
Cholesterol, Span 60 and Tween 60	Cephalexin	Antibacterial	[47]
Cholesterol, Span 40 and Tween 40	Ciprofloxacin	Antibacterial	[48]
Cholesterol, Span 60 and Tween 60			[49]
Cholesterol, span and tween 20	Curcumin	Antinociceptive and anti-inflammatory	[50]
Cholesterol, Span 80, PEG	Daunorubicin and anti-CD123	Treatment of acute myeloid leukemia	[51]
Cholesterol, Span 40 and tween 40	D-limonene	Cancer therapy	[52]
Pluronic L64, Tween 60, EMG 707 Ferrofluid	Doxorubicin	Therapy against chronic myelogenous leukemia	[53]
Pluronic L64, Cholesterol and transferrin		Cancer therapy	[54]
Cholesterol, Span 40 and tween 40	Doxorubicin and Hydrophobin-1	Cancer therapy	[55]
Cholesterol and Span 60	Doxorubicin and N-lauryl glucosamine	Targeted cancer therapy	[56]
Cholesterol, Span 60 and Tween 60	Doxycyclin	Treatment of infection-associated prostate cancer	[57]
Cholesterol and Span 60	Doxycyclin hyclate	Management of ocular diseases	[58]
Cholesterol, Span 60 and phospholipid 90G	Embelin	Diabetes treatment	[59]
Span 40, Span 60, and Brij series 72	Fluconazole	Antifungal treatments	[60]
Cholesterol and Span 60	Flurbiprofen	Anti-inflammatory	[61]
Cholesterol, Span 60 and Tween 65	Gemcitabine and cisplatin	Lung cancer treatment	[62]
Cholesterol, Span 40 and Tween 80	Levofloxacin	Antibacterial	[63]
Cholesterol and Span 60	Linezolid	Antibacterial	[64]
Cholesterol, Span 80 and Tween 80	Methotrexate	Solid tumor treatment	[65]
Span 60, PVA and cremophor RH40			[66]
Cholesterol and glucopyranoside			[67]
Cholesterol and Span 40	Metformin hydrochloride	Avoid Metformin-associated lactic acidosis in the treatment of diabetes mellitus	[68]
Cholesterol and Span 60	Minocyclin	Antibacterial coating of dental implants	[69]
Cholesterol and Tween 60	Moxifloxacin	Antimicrobial	[70]
Cholesterol and tyloxapol	Nevirapine	HIV treatment	[71]
Cholesterol, Span 60 and SolulanC24	N-palmitoylglucosamine	Brain targeting of dynorphin-B	[72]
Cholesterol, Span 60 and PEG	Simvastatin	Against myocardial ischemia/reperfusion injury	[73]
Cholesterol, Span 20 and Span 60		Pediatric transdermal dyslipidemia treatment	[74]
Cholesterol and sorbitan monostearate	Tamoxifen citrate	Incorporated in hydrogel as a pH-responsive drug delivery for breast cancer treatment	[75]
Cholesterol and Span 20	Tamoxifen citrate and doxorubicin	Breast cancer treatment	[76]
Cholesterol, Span60, PEG and TAT peptide	Tenofovir	HIV treatment	[77]
Cholesterol, Span 60 and Tween 40	Timolol maleate	Glaucoma treatment	[78]
Cholesterol and Span 60			[79]
Cholesterol and Span 40			
Cholesterol and Span 60	Timolol maleate and Brimonidine tartrate	Glaucoma treatment	[80]
Cholesterol, Span 60 and Tween 60	Tobramycin	Antibacterial	[81]
Cholesterol, Span 60 and Tween 40	Vancomycin	Antibacterial	[82]
Cholesterol and Span 60		Antibacterial coating for bone plates	[83]
Cholesterol and Tween 40	Zolmitriptan	Migraine treatment	[84]
Cholesterol and Span 60	Chlorotoxin and temozolomide	Targeting and treatment of glyomas	[85]
Cholesterol, Span 60 and PEG	Doxorubicin, curcumin and tLyp-1 peptide	Glioblastoma treatment	[86]
PEG, Tween 80, Octadecylamine	Akt 1 siRNA, Au NPs and Thymoquinone	Treatment of resistance in breast cancer	[87]
Span 80 and PEG	BBIQ [Toll-like receptor (TLR) 7 agonist] and D-1MT [Indoleamine2, 3-dioxygenase (IDO) inhibitor]	Cancer vaccine	[88]
Tween 80 and DTPA-Cl	BMP-7 plasmid	Bone regeneration	[89,90]
Cholesterol and Span 60	CD9 and CD63 tetraspanins	Exosomes immunoassays	[90]
Cholesterol, monopalmitin and Dicetyl phosphate	Influenza antigen	Vaccine and immune response	[91]
Cholesterol, Span 80 and Tween 80	NLS-Mu-Mu fusion protein	Gene delivery	[92]
Tween 60, DOTMA and lycopene	pCMS-EGFP plasmid	Gene delivery to the brain	[93]
Cholesterol, Span 20 and plier-like cationic lipid A (PCL-A)	pDNA or siRNA	Nucleic acid delivery	[94]
DOTMA, Tween 20 and Squalene	pEGFP, pGFP, MC-GFP	Treatment of inherited retinal diseases	[95]
Cholesterol and Span 20	pH (Low) insertion peptide (pHLIP)	Tumor targeting	[96]
Cholesterol, Tween 20 and cationic lipid (N^1^,N^1^-dimyristeroyloxyethyl-spermine)	plasmid DNA-encoding ovalbumin (pOVA)	Skin vaccination	[97]
2,3-di(tetradecyloxy)propan-1-amine cationic lipid, squalene and Tween 80	Plasmid pCMS-EGFP	Delivery of genetic materials to the retina	[98]
Cholesterol and Span 60	Protective antigen (PA) and PA domain 4 (D4) of Bacillus anthracis	prophylaxis against anthrax	[99]
Span 80, DOTAP, TPGS and indocyanine green	siGFP, anti-miR-138	Promote osteogenesis in hMSCs, theranostic applications	[100]
Cholesterol, Tween 85 and DDAB	siRNA	Melanoma treatment	[101]
Cholesterol, Span 20 and plier-like cationic lipid B (PCL-B)	siRNA against anti-apoptotic genes (Mcl-1, Bcl-2 and survivin) and doxorubicin	Breast cancer therapy	[102]
Cholesterol, Span 60 and PEG	siRNA/proteamine and iron superparamagnetic NPs	Breast cancer therapy	[103]
Cholesterol, DOTAP, PEG and Tween 60	siRNA targeted the CDC20 mRNA, doxorubicin and quercetin	Cancer treatment	[104]
Cholesterol and Tween 80	Ciprofloxacin, rifabutin and lignin Ag NPs	Antibacterial	[105]
Cholesterol and Span 80	Curcumin and Ag/Cu NPs	Antibacterial	[106]
Ergosterol, Span 60 and Tween 60	Protamine-condensed DNA and Fe_3_O_4_ NPs	Magnetic properties and cargo-targeted delivery	[107]

**Table 3:** Ethosomes’ drug delivery applications.

Composition	Cargo	Application	Reference
Soy lecithin	5-Aminolevulinic acid	Treatment of hypertrophic scars	[110]
Soy phosphatidylcholine	5-fluorouracil	Treatment of skin cancers	[111,112]
Soy lecithin and cholesterol	Apixaban	Anticoagulant	[113]
Soy phosphatidylcholine	Azelaic acid	Treatment of acne	[114]
Soy phosphatidylcholine and cholesterol	Boswellic acid	Anti-inflammatory	[115]
Phosphatidylcholine	Caffeic acid	Antioxidant	[116]
Soy lecithin	Curcumin and glycyrrhetinic acid	Psoriasis treatment	[117]
DSPE-PEG2000, hydrogenated soy phospholipids and cholesterol	Curcumin, hyaluronic acid and CD44	Psoriasis treatment	[118]
Soy phosphatidylcholine, polyethylenimine and sodium cholate	Doxorubicin and curcumin	Melanoma treatment	[119]
Lecithin and Tween 80	Fenretinide	Chemopreventive for breast cancer	[120]
Soy phosphatidylcholine, cremophor-A25 and chitosan	Ferrous chlorophyllin	Photodynamic therapy for the treatment of squamous cell carcinoma	[121]
Phospholipid 90G	Fisetin	Skin cancers treatment	[122]
Soy phosphatidylcholine	Flurbiprofen	Anti-inflammatory	[123]
Soy phosphatidylcholine	Griseofulvin	Antifungal treatment	[124]
Cholesterol and lecithin	Hyaluronic acid	Transdermal delivery of drugs	[125]
Soy phosphatidylcholine, cholesterol	HRP IgG	Transdermal delivery of vaccines	[126]
Soy phosphatidylcholine, cholesterol and deoxycholic acid	Indomethacin	Treatment of pain and inflammation in rheumatoid arthritis	[127]
Soy lecithin and cholesterol	Luteolin	Anti-tumor activity in hepatocellular carcinoma	[128]
Soy lecithin	Methotrexate	Treatment of psoriasis	[129]
Soy phosphatidylcholine	Methoxsalen	Treatment of vitiligo	[130]
Soy phosphatidylcholine, cholesterol and mannitol	Paenolol	Anti-inflammatory, antidiabetic and pain-relieving	[131]
Soy phosphatidylcholine	Paeoniflorin	Arthritis therapy	[132]
Soy phosphatidylcholine and cholesterol	Phenylethyl resorcinol	Skin Lightening Applications	[133]
Soy phosphatidylcholine, stearylamine and propylene glycol	Resveratrol	Antioxidant	[134]
Phosphatidylcholine	Retinyl palmitate	Acne treatment	[135]
Soy phosphatidylcholine	Sulforaphane	Treatment of skin cancers	[136]
Soy phosphatidylcholine	Terbinafine hydrochloride	Antifungal treatment	[137]
Phospholipid 90G	Thymoquinone	Treatment of acne	[138]
Soy phosphatidylcholine and cholesterol	Thymosin β-4	Wound repair	[139]

**Table 4:** Transfersomes’ drug delivery applications.

Composition	Cargo	Application	Reference
Soy lecithin and Span 80	Aceclofenac	Anti-inflammatory in osteoarthritis	[145]
Soy phosphatidylcholine and Tween 80	Baicalin	Treatment of skin wounds	[146]
Soy phosphatidylcholine and Tween 80	Carvedilol	Prevent skin carcinogenesis	[147]
Phospholipon^®^ 90G and sodium cholate	Cilnidipine	Treatment of hypertension	[148]
Soy phosphatidylcholine	Deferoxamine	Treatment of pressure ulcers	[149]
DPPC, cholesterol, TPGS and folate	Docetaxel	Treatment of glioblastoma multiforme	[150]
Soy phosphatidylcholine and sodium cholate	Epigallocatechin-3-gallate and hyaluronic acid	Anti-aging and antioxidant	[151]
Soy phosphatidylcholine and Tween 80	Eprosartan mesylate	Treatment of hypertension	[152]
Soy phosphatidylcholine and Span 80	Genistein (GEN-TF2)	Therapeutic or preventive strategy against neurodegenerative diseases	[153]
Soy lecithin and Sodium Lauryl Sulphate	Ivabradine HCl	Treatment of stable angina pectoris	[154]
Soy lecithin and Tween 80	Mangiferin	Treatment of skin wounds	[155]
Phospholipon (PL) 90H and Span 60	Natamycin	Antifungal	[156]
Phospholipon 90 G^®^ and sodium cholate	Pentoxifylline	Treatment of intermittent claudication and chronic occlusive arterial diseases	[157]
Lecithin and Tween 20/80	Resveratrol	Antioxidant	[158]
Soy phosphatidylcholine, Tween 80 and ceramide III	Retinyl palmitate	Antioxidant	[159]
Soy phosphatidylcholine and emu oil	Tamoxifen	Transdermal therapy for breast cancer	[160]
Soy lecithin and Tween 80	Taxifolin	Antioxidant	[161]
Soy phosphatidylcholine and Tween 80	Tocopherol	Antioxidant	[162]
Soya lecithin and Tween 80	Zolmitriptan	Migraine treatment	[163]
Soy lecithin and sodium deoxycholate	Human growth hormone	Transdermal hormone delivery	[164]
Egg phosphatidylcholine, stearylamine and Tween 20	PnPP-19 peptide	Treatment of erectile dysfunction	[165]

**Table 6:** Ufasomes’ drug delivery applications.

Composition	Cargo	Application	Reference
Cholesterol and oleic acid	Cinnarizine	Antihistaminic activity	[184]
Phosphatidylcholine from soy and oleic acid	Minoxidil	Hypertension treatment	[185]
Phosphatidylcholine from soy oleic and linoleic acid	Oleuropein	Antioxidant activity	[183]
Oleic acid and tea tree oil	Oxiconazole	Candida albicans treatment	[186]
Glyceryl oleate	Terbinafine hydrochloride	Candida albicans treatment	[187]

**Table 7:** Phytosomes’ drug delivery applications.

Composition	Cargo	Application	Reference
Phosphatidylcholine	Abutilon indicum and Piper longum	Hepatoprotective effect	[191]
Phosphatidylcholine	Annona muricata L. aqueous extract	Treatment of major depressive disorders	[192]
Milk phospholipids	Ascorbic acid and α-tocopherol	Antioxidative	[193]
Phosphatidylcholine	Berberine	Diabetes treatment	[194]
Phosphatidylcholine	Chicoric acid and chlorogenic acid from the Echinacea plant	Antioxidant activity	[195]
Egg phospholipid	Chrysin	Diabetes treatment	[196,197]
Lecithin	Diosgenin	Lung cancer treatment	[198]
Phosphatidylcholine	Diosmin	Vascular protection activity	[199]
Phosphatidylcholine and piperine	Domperidone	Anti-emetic effect	[200]
Lecithin	Ethanolic extract of leaves of Bombax ceiba	Hepatoprotective effect	[201]
Lipoid^®^ S45	Flavonoids from Citrullus colocynthis, mormodica balsamina l. and mormodica dioica roxb.	Diabetes treatment	[202]
Lipoid^®^ S100 and Phosal^®^ 75 SA	Genistein	Hepatocellular carcinoma treatment	[203]
Soy Hydrogenated Phosphatidylcholine	Icariin	Treatment of ovarian cancer	[204]
Phosphatidylcholine	Momordica charantia extract	Hypoglycemic effect	[205]
DPPH and phosphatidylcholine	Persimmon extract	Antioxidative	[206]
Phosphatidylcholine	Propolis	Antioxidant activity	[207]
DPPC	Rutin	Antioxidant for the prevention of liver inflammation	[208]
Lecithin	Silymarin	Antioxidant, hepatoprotective and anticancer activity	[209]
Lecithin	Taxifolin rich fraction of Cedrus deodara bark extract	Breast cancer treatment	[210]
Soy Hydrogenated Phosphatidylcholine	Thymoquinone	Lung cancer treatment	[211]
Phosphatidylcholine	Tripterine	Cancer treatment	[212]
Lipoid S100	Tripterine and selenium	Arthritis treatment	[213]
Phosphatidylcholine	Umbelliferone	Photo-protective and antioxidant activity	[214]

**Table 8:** Catanionic vesicles’ drug delivery applications. In the composition column, C is the cationic and A the anionic compound.

Composition	Cargo	Application	Reference
C: ester functionalized morpholinium and imidazolium-based surface active ionic liquids A: sodium butyrate	Curcumin	Antimicrobial activity	[219]
C: CTAB A: SDS		Lung cancer treatment	[220]
C: CTAB A: SDS	Diclofenac sodium	Anti-inflammatory	[221]
Serine-based surfactants C: 16Ser A: 8-8Ser	Doxorubicin	Cancer treatment	[222]
C: 4-cholesterocarbonyl-4′-(N,N,N-triethylamine butyloxyl bromide) azobenzene A: SDS		Antioxidant activity	[223]
C: CTAT A: sodium dodecylbenzenesulfonate	Francisella tularensis lisate	Tularemia vaccine	[224]
C: benzyldimethylhexadecyl ammonium chloride A: sodium 1,4-bis (2-ethylhexyl) sulfosuccinate	Insulin	Diabetes treatment	[225]
C: Azobenzene-based surfactant A: sodium dodecylbenzenesulfonate	Paclitaxel and Bcl-2 siRNA	Breast cancer treatment	[226]
C: hexadecyltrimethyl ammonium copper trichloride A: SDS	Toluidine blue and Rose Bengal	Antimicrobial Photodynamic Therapy against Escherichia coli	[227,228]
C: CTAC A: SDS	Trans-resveratrol	Antioxidant and radical scavenging activity	[229]
C: arginine-based surfactants A: sodium laurate, sodium myristate and 8-SH		Antimicrobial and antibiofilm activity	[218]
C: cetalkonium chloride A: diclofenac sodium, flurbiprofen sodium or naproxen sodium		Anti-inflammatory drug release from contact lenses	[230]
C: chlorambucil prodrug A: sodium bis (2-ethylhexyl) sulfosuccinate		Cancer treatment	[231]
C: Cytarabine hydrochloride A: Sericin protein surfactant		Cancer treatment	[232]
C: CTAT A: sodium dodecylbenzenesulfonate		Extraction of cell surface components of Neisseria gonorrhoeae into the leaflet of the vesicles to create artificial pathogens for vaccines	[233]
C: doxorubicin A: gemini surfactant		Cancer treatment	[234]
C: DTAB A: dioctyl sulfosuccinate sodium salt		Drug delivery for cystic fibrosis	[235]
C: hexamethylene-1,6-bis (dodecyldimethylammonium) dibromide A: diclofenac sodium		Antimicrobial activity	[236]
C: methylimidazolium- or pyridinium-based surface active ionic liquids A: sodium N-lauroyl sarcosinate		Antimicrobial activity	[237]
C: methylimidazolium- or pyridinium-based surface active ionic liquids A: sodium bis(2-ethyl-1-hexyl) sulfosuccinate		Antimicrobial activity	[238]
C: NαNω-Bis(Nαcaproylarginine) α,ω-propyldiamide A: Lichenysin		Antimicrobial and antifungal activity	[239]
C: N(π), N(τ)-bis(methyl)-L-Histidine tetradecyl amide A: lysine-based surfactant Nα-lauroyl-Nεacetyl lysine or sodium myristate		Antimicrobial activity	[240]
C: N-dodecylamino-1-deoxylactitol A: ketoprofen		Anti-inflammatory activity	[241]

**Table 9:** Extracellular vesicles’ drug delivery applications.

Parental cell	Cargo	Application	Reference
EVs from HEK293T cells	Angiotensin converting enzyme II (ACE2)	Protect from SARS-CoV-2 infection by competitively bound to virus against host cells	[253]
Milk-derived exosomes	Anthocyanidins	Anti-proliferative and anti-inflammatory in lung cancer	[254]
Exosomes from breast and colorectal cancer cells	Aspirin	Cancer therapy	[255]
Exosomes from MIN-6 cells	BAY55-9837	Increase insulin production for type 2 diabetes mellitus	[256]
Exosomes from macrophages	Berberine	Spinal cord injury treatment	[257]
EVs from human umbilical cord mesenchymal stem cells	Cannabidiol	Increase the therapeutic efficacy of doxorubicin in triple negative breast cancer	[258]
Exosomes from umbilical cord-derived macrophages	Cisplatin	Ovarian cancer cells treatment	[259]
EVs from macrophages	Curcumin	Neuroprotection and ischemia-reperfusion injury treatment	[260]
		Inhibit the phosphorylation of Tau protein	[261]
Exosomes from mesenchymal stem cells		Attenuate the progression of osteoarthritis	[262]
EVs from HEK293 cells		Myocardial infarction treatment	[263]
Exosomes from bone marrow-derived mesenchymal stem cells		Cerebral ischemia treatment	[264]
Exosomes from HEK293 cells	Curcumin and RAGE-binding peptide	Acute lung injury treatment	[265]
EVs from smooth muscle cells	Cystatin C	Protection and healing of the nervous system in different neurotoxic conditions	[266]
Exosomes from lung cancer	Docetaxel	Non-small cell lung cancer treatment	[267]
Exosomes from cervical cancer		Cervical cancer treatment	[268]
Exosomes from blood samples	Dopamine	Parkinson’s disease treatment	[269]
EVs from macrophages	Doxorubicin	Metastatic ovarian cancer treatment	[270]
Exosomes from mesenchymal stem cells		Colorectal cancer treatment	[271]
Exosomes from human glioma		Glioma treatment	[272]
Milk-derived exosomes		Cancer treatment	[273]
Exosomes from HEK293 cells		Cancer treatment	[274]
Exosomes from bone marrow-derived mesenchymal stem cells		Osteosarcoma treatment	[275]
Exosomes from colon cancer		Colorectal cancer treatment	[276]
Exosomes from human breast and ovarian cancer		Breast and ovarian cancer treatment	[277]
Exosomes from macrophages	Edaravone	Permanent middle cerebral artery occlusion treatment	[278]
Exosomes from human fetal lung fibroblasts	Erastin	Triple-negative breast cancer therapy	[279]
Exosomes from pancreatic cells	Gemcitabine	Pancreatic cancer treatment	[280]
EVs from human plasma	Imperialine	Non-small cell lung cancer treatment	[281]
EVs from human umbilical vascular endothelial cells	Meta-tetra(hydroxyphenyl) chlorine	Cancer photodynamic therapy	[282,283]
EVs from fibroblast cells	Methotrexate	Glioblastoma treatment	[284]
Exosomes from embryonic stem cells	Paclitaxel	Glioblastoma treatment	[285]
Exosomes from mesenchymal stem cells		Carcinoma treatment	[286]
EVs from gingival mesenchymal stromal cells		Cancer treatment	[287,288]
Exosomes from macrophages		Pulmonary metastases treatment	[289]
Milk-derived exosomes		Lung cancer treatment	[290]
EVs from bone marrow mesenchymal stromal cells		Malignant pleural mesothelioma treatment	[291]
Exosomes from macrophages		Multiple drug-resistant cancer treatment	[292]
EVs from lung cancer cells	Paclitaxel and oncolytic virus	Primary and metastatic cancer treatment	[293]
EVs from neutrophil-like cells	Piceatannol	Alleviated acute lung inflammation/injury and sepsis induced by lipopolysaccharide	[294]
Exosomes from plasma	Quercetin	Relieve symptoms of Alzheimer’s disease by inhibiting phosphorylation of Tau and reducing the formation of insoluble neurofibrillary tangles	[295]
Exosomes from human ovarian cancer	Triptolide	Ovarian cancer treatment	[296]
Mannosylated exosomes from macrophages	Vancomycin and lysostaphin	Eradication of intracellular quiescent MRSA	[297]
Exosomes from fibroblasts	WNT3A	Repair of osteochondral defects	[298]

**Table 10:** Extracellular vesicles’ inorganic NPs delivery applications.

Parental Cell	Cargo	Application	Reference
Exosomes from human hepatocarcinoma	Doxorubicin-loaded biomimetic porous silicon NPs	Cytotoxicity against bulk cancer cells and cancer stem cells	[300]
Grapefruit EVs	Doxorubicin-loaded heparin-based NPs	Glioma treatment	[301]
Exosomes from melanoma cells	Gold NPs	Cancer treatment	[302]
Exosomes from HEK293T cells		Blood-brain barrier penetration and brain disorders future treatments	[303]
Exosomes from bone marrow mesenchymal stromal cells		Neuroimaging for various brain disorders	[304]
Exosomes from mesenchymal stem cells			[305]
Exosomes from breast cancer cells	Gold iron oxide hybrid NPs	MRI contrast agent and photodynamic therapy	[306]
Exosomes from mesenchymal stem cells	Iron oxide NPs	Myocardial infarction treatment	[307]
		Wound repair	[308]
		Increase activation and migration ability of macrophage	[309]
		Tumor cell ablation via magnetically induced hyperthermia	[310]
EVs from human umbilical vascular endothelial cells		Photodynamic and hyperthermia therapy of prostate cancer	[311]
Exosomes from macrophages	Laurate-functionalized Pt(IV) prodrug, human serum albumin, and lecithin NPs	Breast cancer and metastatic breast cancer lung nodules treatment	[312]
Exosomes from lung adenocarcinoma cells	Metal-organic framework	Detection of the ATP level in living cancer cells, providing an efficient tool for the cell metabolism study	[313]
Exosomes from triple negative breast cancer cells		Delivery of anticancer compounds	[314]
Exosomes from HeLa cells			[315]
Exosomes from lung cancer or glioma	Palladium nanosheet	Deliver catalytic cargo directly to cancer cells	[316]
Exosomes from triple negative breast cancer cells	PLGA NPs	Cancer therapy	[317]
Exosomes from lung carcinoma cells			[318]
EVs from Staphylococcus aureus		Intracellular delivery of antibiotics for intracellular pathogen-associated complications treatment	[319]
Exosomes from breast cancer	Quantum dots of vanadium carbide	Cancer photothermal therapy	[320]
Exosomes from hepatocellular carcinoma	Silver and iron NCs	Cancer bioimaging	[321]
Exosomes from macrophages	SPIONs and curcumin	Synergistic antitumor therapy in gliomas	[322]
Exosomes from plasma	Superparamagnetic magnetite colloidal nanocrystal clusters	Cancer treatment	[323]
EVs from KB cells	Zinc oxide NCs	Cancer treatment	[324]

**Table 11:** Extracellular vesicles’ nucleic acids delivery applications.

Parental Cell	Cargo	Application	Reference
Microvesicles from breast cancer cells	Minicircle DNA encoding a thymidine kinase /nitroreductase fusion protein	Breast cancer therapy	[335]
EVs from mice melanoma cells	Plasmid DNA coding for ESAT-6	Promote antitumor activity of dendritic cells	[336]
EVs from human brain endothelial cells and macrophages	Plasmid DNA encoding for brain-derived neurotrophic factor	Protection of the brain endothelium increasing endothelial ATP levels	[337]
EVs from macrophage cells	Tripeptidyl peptidase-1-encoding plasmid DNA	Lysosomal storage disorder, Neuronal Ceroid Lipofuscinoses 2 (CLN2) or Batten disease treatment	[338]
EVs from red blood cells	Anti-miR-125b ASOs and Cas9 mRNA	Cancer treatment	[339]
Exosomes from mouse neuronal cells	miR-21-5p	Suppression of autophagy after a traumatic brain injury	[340]
EVs from frozen human plasma	miR-31 and miR-451a	Promoted apoptosis of hepatocellular carcinoma	[341]
Exosomes from human bone marrow mesenchymal stem cells	miR-101-3p	Oral cancer treatment	[342]
Exosomes from bone marrow mesenchymal stem cells	miR-124	Promote neurogenesis after ischemia	[343]
EVs from human adipose tissue-derived mesenchymal stromal/ medicinal signaling cells	miR-125b	Inhibits hepatocellular carcinoma proliferation	[344]
Exosomes from normal intestinal epithelial FHC cells	miR-128-3p	Increase chemosensitivity of oxaliplatin-resistant colorectal cancer	[345]
Exosomes from HKT293T cells	Curcumin, saponin, MiR-143	Engineered exosomes for anti-HIV agents delivery to solid tissues	[346]
Exosomes from human umbilical cord mesenchymal stem cells	miR-145-5p	Inhibit adenocarcinoma progression	[347]
EVs from bone-marrow mesenchymal stem cells	miR-146a	Ulcerative colitis treatment	[348]
EVs from human mesenchymal stromal cells	miR-146a-5p	Prevent group 2 innate lymphoid cells -dominant allergic airway inflammation	[349]
Exosomes from human umbilical cord mesenchymal stem cells	miR-148b-3p	Suppress breast cancer progression	[350]
Exosomes from mesenchymal stem cells	miR-199a	Inhibit the growth of glioma by down-regulating AGAP2	[351]
Exosomes from endothelial progenitor cells	miR-210	Protect endothelial cells against hypoxia/ reoxygenation injury improving mitochondrial function	[352]
EVs from mesenchymal stem cells	miR-210	Promote angiogenesis in myocardial infarction	[353]
EVs from bone mesenchymal stem cells	miR-216a-5p	Promote the proliferation of chondrocytes in osteoarthritis	[354]
EVs from human umbilical cord mesenchymal stem cells	miR-302a	Therapy of endometrial cancer	[355]
EVs from mesenchymal stem cells	miR-379	Therapy for metastatic breast cancer	[245]
EVs from adipose tissue-mesenchymal stromal cells	miR-424-5p	Therapy for triple negative breast cancer	[356]
Exosomes from HEK-293T cells	miR-497	Inhibit lung cancer growth and angiogenesis	[357]
Exosomes from CRC cells	miR-567	Reverse chemoresistance to Trastuzumab in breast cancer	[358]
EVs from HEK-293T cells	miR-1252-5p	Downregulation of heparanase to enhance the chemosensitivity to Bortezomib in multiple myeloma	[359]
EVs from HEK-293T cells	miRNA-21	Myocardial infarction treatment	[360]
Exosomes from breast cancer	miRNA-126	Inhibit the formation of lung cancer metastasis	[361]
EVs from glioblastoma stem-like cells	miRNA-139	Downregulation of glioblastoma	[362]
Exosomes from mesenchymal stem cells	miRNA-584-5p	Gliomas treatment	[363]
Exosomes 293F cells	mRNA	SARS-CoV-2 vaccine	[364]
Exosomes from HEK-293T cells	Catalase mRNA	Attenuated neurotoxicity and neuroinflammation in Parkinson’s disease	[365]
EVs from HEK-293T cells	Cytosine deaminase fused to uracil phosphoribosyltransferase mRNA	Glioblastoma treatment	[366]
EVs from HEK-293T cells	HChrR6 mRNA	Convert CNOB into MCHB for the treatment of cancers	[367]
Exosomes from mesenchymal stem cells, dendritic cells or HEK-293T cells	PTEN mRNA	Restore tumor-suppressor function in PTEN deficient gliomas	[368]
EVs from non-pigmented ciliary epithelium cells	anti-fibrotic (SMAD7) siRNA	Lower intraocular pressure in primary open-angle glaucoma	[369]
Exosomes from autologous breast cancer cells	Cationic bovine serum albumin conjugated siS100A4	Suppress postoperative breast cancer metastasis	[370]
EVs from murine neuroblastoma cell line and dendritic cells	Cholesterol-conjugated siRNAs	Human antigen R silencing for cancer treatment	[334]
Exosomes from HEK-293T cells	c-Met siRNA	Reverse chemoresistance to cisplatin in gastric cancer	[371]
Exosomes from HEK-293T cells	Hepatocyte growth factor (HGF) siRNA	Inhibitory effect on tumor growth and angiogenesis in gastric cancer	[372]
EVs from mesenchymal stem cells derived from umbilical cord Wharton’s jelly	Hydrophobically modified asymmetric siRNAs conjugated with cholesterol	Huntingtin silencing in neurons	[333]
Exosomes from glioblastoma cells			[373]
Exosomes from human neuroblastoma cells	Heat shock protein-27 (HSP27) siRNA	Decrease of cell differentiation toward mature neuron in neuroblastoma	[374]
Exosomes from urine-derived induced pluripotent stem cells	ICAM-1 siRNA	Alleviating inflammation of pulmonary microvascular endothelial cells	[375]
Exosomes from HEK-293T cells	KRAS siRNA	Inhibition of tumor growth	[376]
EVs from astrocytes	LincRNA-Cox2 siRNA	Lipopolysaccharideinduced microglial proliferation for treatment of CNS disorders	[377]
Exosomes from mesenchymal stem cells	PTEN siRNA	Promote recovery for spinal cord injury individuals	[378]
EVs from red blood cells	P65 and Snai1 siRNA	Inhibit renal inflammation and fibrosis for acute kidney injury treatment	[379]
EVs from HEK-293T cells	RAGE siRNA	Attenuated inflammation in myocarditis	[380]
Exosomes from bone-marrow-derived mesenchymal stem cells	siGRP78	Suppress Sorafenib resistance in hepatocellular carcinoma	[381]
Exosomes from bovine milk	siKRAS	Lung tumor treatment	[382]
EVs from different cell lines	siRNA	Reducing the therapeutic dose of siRNA for different pathologies	[383]
EVs from human umbilical cord mesenchymal stem cells	siRNA-ELFN1-AS1	Inhibit colon adenocarcinoma cells proliferation	[384]
Exosomes from normal human foreskin fibroblast	siRNA or short hairpin RNA specific to oncogenic Kras^G12D^	Pancreatic ductal adenocarcinoma treatment	[385]
Exosomes from HEK-293T cells	Transient receptor potential polycystic 2 (TRPP2) siRNA	Reduce the epithelial-mesenchymal transition in pharyngeal squamous carcinoma	[386]
Exosomes from brain endothelial bEND.3 cells	Vascular endothelial growth factor (VEGF) siRNA	Knockdown of VEGF in brain cancer cells	[387]
Exosomes from HEK-293T cells	Different viral products including Ebola Virus VP24, VP40 and NP, Influenza Virus NP, Crimean–Congo Hemorrhagic Fever NP, West Nile Virus NS3, and Hepatitis C Virus NS3	Exosomes-based vaccines	[388]

## Data Availability

Data presented in this manuscript is available from corresponding author upon reseanable requests.
